# Non-technical skills training for Nigerian interprofessional surgical teams: a cross-sectional survey

**DOI:** 10.1186/s12909-024-05550-8

**Published:** 2024-05-16

**Authors:** Barnabas Tobi Alayande, Callum Forbes, Paul Kingpriest, Adeyinka Adejumo, Wendy Williams, Felix Wina, Christian Agbo Agbo, Bamidele Omolabake, Abebe Bekele, Bashiru O Ismaila, Fiona Kerray, Augustine Sule, Egide Abahuje, Jamie M. Robertson, The Non-technical Skills for Surgery Nigeria Group, Tosin Abah, Akims Shattah, Linus Hapiyati Homoweto, John Onyeji, Joseph Okoko, Joshua Sule, Steven Yule, Robert Riviello, Mercy Isichei

**Affiliations:** 1 Center for Equity in Global Surgery, University of Global Health Equity, Kigali, Rwanda; 2 Department of Surgery, Jos University Teaching Hospital, Jos, Nigeria; 3 Program in Global Surgery and Social Change, Harvard Medical School, Boston, MA United States of America; 4 Surgical Equity Research Hub, Jos, Nigeria; 5 Federal Medical Centre, Keffi, Nasarawa State Nigeria; 6 Center for Surgery and Public Health, Brigham and Women’s Hospital, Boston, MA United States of America; 7 Department of Surgery, Bingham University Teaching Hospital, Jos, Nigeria; 8 Department of Surgery, Benue State University Teaching Hospital, Markudi, Nigeria; 9 Department of Clinical Surgery, The University of Edinburgh, Edinburgh, UK; 10 University of Rwanda, Kigali, Rwanda; 11 Department of Surgery, Northwestern University, Evanston, Ilinois United States of America; 12 Department of Surgery, Brigham and Women’s Hospital, Boston, MA United States of America; 13 The Faith Alive Foundation, Jos, Nigeria

**Keywords:** Non-technical skills for surgery, Variable resource contexts, Nigeria

## Abstract

**Introduction:**

Non-technical skills (NTS) including communication, teamwork, leadership, situational awareness, and decision making, are essential for enhancing surgical safety. Often perceived as tangential soft skills, NTS are many times not included in formal medical education curricula or continuing medical professional development. We aimed to explore exposure of interprofessional teams in North-Central Nigeria to NTS and ascertain perceived facilitators and barriers to interprofessional training in these skills to enhance surgical safety and inform design of a relevant contextualized curriculum.

**Methods:**

Six health facilities characterised by high surgical volumes in Nigeria’s North-Central geopolitical zone were purposively identified. Federal, state, and private university teaching hospitals, non-teaching public and private hospitals, and a not-for-profit health facility were included. A nineteen-item, web-based, cross-sectional survey was distributed to 71 surgical providers, operating room nurses, and anaesthesia providers by snowball sampling through interprofessional surgical team leads from August to November 2021. Data were analysed using Fisher’s exact test, proportions, and constant comparative methods for free text responses.

**Results:**

Respondents included 17 anaesthesia providers, 21 perioperative nurses, and 29 surgeons and surgical trainees, with a 95.7% survey completion rate. Over 96% had never heard of any NTS for surgery framework useful for variable resource contexts and only 8% had ever received any form of NTS training. Interprofessional teams identified communication and teamwork as the most deficient personal skills (38, 57%), and as the most needed for surgical team improvement (45, 67%). There was a very high demand for NTS training by all surgical team members (64, 96%). The main motivations for training were expectations of resultant improved patient safety and improved interprofessional team dynamics. Week-long, hybrid training courses (with combined in-person and online components) were the preferred format for delivery of NTS education. Factors that would facilitate attendance included a desire for patient safety and self-improvement, while barriers to attendance were conflicts of time, and training costs.

**Conclusions:**

Interprofessional surgical teams in the Nigerian context have a high degree of interest in NTS training, and believe it can improve team dynamics, personal performance, and ultimately patient safety. Implementation of NTS training programs should emphasize interprofessional communication and teamworking.

## Background

Non-technical skills (NTS) are defined as a constellation of cognitive and social skills, demonstrated by teams and individuals, needed to reduce error, and to improve human performance in complex systems [1]. Derived originally from high-risk industries like aviation, aerospace, nuclear, explosive, military, and high-speed sports, these skills are essential in surgical care [2]. These skills can enhance the way surgical teams carry out surgery [1].

Failure of NTS such as situation awareness, decision making, leadership, communication and teamwork has been shown to contribute to up to half of all intra-operative errors [3]. Often considered soft skills, these competencies are many times overlooked in both formal and informal clinical training - particularly in low resource contexts where emphasis is placed on technical skills [4]. However, published literature shows that failure of NTS is a significant cause of adverse events in over 50% of all fatal medical accidents [5]. The consequence of not teaching NTS is at the heart of errors, sentinel events and near misses compromising a patient safety culture [6].

In addressing the absence of reliable ways to teach NTS, the Royal College of Surgeons, Edinburgh, and the University of Aberdeen developed the Non-Technical Skills for Surgeons (NOTSS) framework [1]. NOTSS refers to a behaviour rating tool grounded in a skills taxonomy that permits a valid and reliable observation and assessment of situation awareness, decision making, leadership, communication, and teamwork [7]. Considered by some as the gold standard for NTS, NOTSS has been extensively used across the world to prevent or mitigate surgical errors [8].

Even though up to 76 different published tools have been used to measure NTS in seven distinct areas of clinical practice, NTS training has been largely restricted to the high-income context [8, 9]. Through a mixed methods approach involving Rwandan surgeons, anaesthetists, and nurses, the NOTSS behaviour rating system was modified for use in a variable resource context [4]. Non-Technical Skills for Surgery in Variable Resource Context (NOTSS-VRC) is targeted to address variability in resources, staff, systems support, and language frequently encountered by surgical teams in low- and middle-income countries [10]. The key modifications in NOTSS-VRC have been in the inclusion of contextual indicative behaviours that address this variability [4, 10, 11]. Other NTS courses have been designed for low-resource settings such as the Vital Anaesthesia Simulation Training (VAST) and SAFE Operating Room Course [12–14].

Although Nigeria and Rwanda are both sub-Saharan African countries and share some commonalities, they are different in regional location (West versus East Africa), working languages of healthcare teams, health system organization, specific kinds of resource variability, and availability of NTS training. Currently, there is no identified programme holistically offering training or continuing medical education using this framework for surgery, obstetrics, trauma, anaesthesia, or nursing in West Africa. This survey was designed to gather input from health professionals and trainees working in North-Central Nigeria on the need for NTS for surgery specific to surgeons, perioperative nurses, and anaesthetists.

Before modifying or implementing any NTS training program, it is important to assess the interest, preparedness, and training preferences of the local Nigerian workforce. A bespoke survey was developed to evaluate this by our multidisciplinary group of local researchers collaborating with the NOTSS global team consisting of clinical educators, surgeons, anaesthetists, a clinical psychologist, nurses, and surgical safety and human factors trainees [11]. A well-designed NTS program should be highly contextualized and meet the needs of the target healthcare workers [15]. The focus of the survey was to identify trained surgical providers’ current exposure to NTS and highlight the perceived needs and preferences of local surgical teams to guide the design of training modules on NTS for the Nigerian variable resource context.

## Methods

The survey design adhered to the Checklist for Reporting Results of Internet E-Surveys (CHERRIES) [16]. It was primarily a quantitative survey with a section for qualitative written responses. The voluntary survey was designed by a local team of surgical providers with training in NOTSS-VRC from Rwanda. Mentorship for the design was provided by the multidisciplinary and trans-sectoral NOTSS Global team. The survey was targeted at the 133 surgical providers, anaesthesia providers, and perioperative nurses working within six purposively selected institutions in North-Central Nigeria, spanning a wide variety of facility type and governance. These facilities included a non-profit secondary facility with focus on HIV-related surgical care, a private tertiary health facility, a specialist hospital, a state government-owned teaching hospital, a non-teaching federal medical centre, and a federal government-owned teaching hospital. These facilities were selected for their high surgical volumes and the presence of a multidisciplinary and interprofessional surgical team. Using a 95% confidence interval and an 8% margin of error, a representative sample of 71 out of 133 was selected through a convenience, stratified snowball technique at each institution, starting from the head of surgery, the lead perioperative nurse, or the lead anaesthesia provider. We elected to use convenience sampling to select surgical team leads based on their accessibility and availability to the researchers. Rather than being drawn at random from a larger population, in this strategy, participants were picked because they are easily available to the research team and would be able to influence propagation of the survey as leaders. We then stratified these leaders by their speciality into nursing team leads, surgical heads of department, and anaesthesia heads of department so that all cadres within the operating room are represented. The rational for the stratified snowball method was that these leaders would be able to identify and connect us to their peers who worked in interprofessional teams at the selected hospitals more effectively.

Ethical clearance for the NTS study was obtained from the Jos University Teaching Hospital, Nigeria Institutional Review Board (JUTH/DCS/IREC/127/XXXI/2277). Informed consent was obtained from participants prior to taking the approximately 7-minute survey. Participants were informed of the purpose of the survey, introduced to the primary investigator, and told the approximate length of time needed to fill the survey prior to consent. The survey consisted of close-ended questions (for quantitative analysis) and a free text response component (for qualitative analysis). The role of the individual on the surgical team, previous knowledge about NTS for surgery and NOTSS-VRC, and details of any prior training were collected. Respondents’ perceptions of the importance of constructs of situation awareness, decision making, leadership, communication and teamwork in the Nigerian context, and their perception of the single most important NTS for personal and team improvement were identified. The survey also identified interest in formal training in NTS, the perceived benefits of NTS. In addition, the survey collected responses on the ideal duration and format for a NTS training program (in person versus online versus blended). A free text section collected hindrances and enabling factors for participating in NTS training.

Names, demographic information, and institutional affiliation were not collected. No personal information was collected or stored. Information was collected using a restricted Google form, and unauthorized access was prevented by limiting editing rights to three investigators. Respondents were able to review and change their answers using a back button. Access to collected data was only permitted on password-protected computer devices. The survey was developed through an iterative process, and the usability and technical functionality of the electronic questionnaire was pre-tested before dissemination. The open survey was not advertised online but was disseminated to surgical, obstetrics, anaesthesia, and perioperative nursing leads at target facilities. The survey was limited to clinically active participants. Initial contact with potential participants was made on WhatsApp, and the forms required web-based data entry. No cookies, IP address check, or automated log file analysis was performed; however, data were cleaned manually and examined for double entries and other inconsistencies including submissions with atypical timestamps.

Surveys were administered through an electronic, web-based, single-page Google form with 19 questionnaire items, and responses were automatically captured. No incentives were provided to any participants. Data were collected from August 9 to October 6, 2021. Items and questionnaires were not randomized or alternated. A post-submission completeness check was carried out, finding that 6 respondents had incomplete entries. The response rate was 95.7% (*n* = 68; *N* = 71); and the completion rate (the number of people submitting the last questionnaire page, divided by the number of people who agreed to participate or submitted the first survey page) was also 95.7% (*n* = 68; *N* = 71). Any questionnaires with less than 50% completion, or with missing demographics (*n* = 4) were excluded from analysis. Questionnaire items were not weighted, and propensity scores were not applied to adjust for any sample. Analysis was carried out in R software version 4.1.0 [17] using proportions and Fisher’s exact test. We analysed free text written responses to identify barriers and enhancers to NTS training in the context and generated themes using the qualitative constant comparative method as described by Glaser [18]. Responses were quantised by theme and presented by frequency and percentage alongside quotes of sample phrases. We used group open coding involving one investigator and a research assistant (BA and PK) and resolved disagreements by discussion. Qualitative analysis was carried out using a grounded theory qualitative approach and a constructivist research paradigm and a convergent (parallel) design [19]. The free text data were analysed using topic detection/categorisation technique which employs grouping or bucketing of similar themes relevant for the project [19]. No sub-categories were identified. Inductive qualitative analysis was carried out [18]. For the two open-ended questions from the survey, we categorized the text into a number of similar themes in an inductive manner [18, 19].

## Results

There was a total of 67 respondents, which included 17 anaesthesia providers (25.4%), 21 perioperative nurses (31.3%), and 29 surgeons and surgeons-in-training (43.3%).

Overall, there was poor awareness of NTS across all specialties (Table 1), with only 32 of 67 (47.8%) having heard of NTS use in surgery. This shortfall in awareness was most evident amongst the anaesthesia providers with only 6 of 17 (35.3%) having previously heard of NTS, compared with 13 of 29 (44.8%) of surgical providers, and 13 of 21 (61.9%) of perioperative nursing staff. Similarly, awareness of NOTSS-VRC training was low with only 14 (21.2%) having previously heard of any NTS course adapted for variable resource contexts. This was particularly true of surgeons/surgeons-in-training relative to other professions (*p* = 0.022), as only 2 (6.9%) had prior knowledge of the course. Only 6 respondents (9.1%), none of whom were surgeons, had previously attended a NTS training program (*p* = 0.031). The NTS training courses that respondents specified that they had attended previously were not purely NTS training, as they were exclusively local hospital or university-based training sessions rather than internationally recognised training courses. One respondent identified a ‘handling and maintenance of minimally invasive instruments’ training course as containing NTS.

**Table 1:** Awareness and interest in non-technical skills training among perioperative nurses, anaesthesia providers and surgeons in North-Central Nigeria

	Total		Anaesthesia providers *N* = 17		Surgeons* N = 29		Perioperative Nurses *N* = 21		*p*- value**
**Awareness and Interest**	n	%	n	%	n	%	n	%	
Respondents aware of NTS*** for surgery (*N* = 67)	32	47.8	6	35.3	13	44.8	13	61.9	*p* = 0.248
Respondents aware of NOTSS-VRC**** (*N* = 66)	14	21.2	4	25.0	2	6.9	8	38.1	*p* = 0.022
Have had previous NTS training (*N* = 66)	5	7.6	2	12.5	0	0	3	14.3	*p* = 0.031
Express interest in NTS training (*N* = 66)	65	95.6	17	100.0	27	93.1	20	95.2	*p* = 0.549
Believe that improved NTS will improve patient safety	66	98.5	17	100.0	29	100.0	20	95.2	*p* = 0.329

*Includes surgical trainees

**2-sided Fisher’s exact test

***NTS non-technical skills

****NOTSS-VRC Non-Technical Skills for Surgery in Variable Resource Contexts

When asked to rate each of the components of NTS in terms of importance, all categories were rated predominantly as ‘very important’ (86.6–91.0%). None of the NTS categories were rated as ‘not important’ by any of the respondents. In ranking the four categories (Table 2), overall, most respondents (38, 56.7%) ranked ‘communication and teamwork’ as the one they would most personally like to learn about and ‘situational awareness’ as the least. This held true across specialties with no statistically significant difference between groups’ ranking of the categories. In addition, respondents perceived that ‘communication and teamwork’ were most needed for interprofessional team improvement in their context. Deviating from other surgeons and anaesthesia providers, nursing staff ranked decision making as less important than leadership; however, this was not statistically significant (Fisher’s exact *p*-value = 0.055).

**Table 2:** Ranking of non-technical skills for surgery categories based on perceived training need

	Total		Anaesthesia providers		Surgeons and trainees		Nurses		*p*-value
	*n*	%	*n*	%	*n*	%	*n*	%	
Non-technical skills most needed for personal improvement									0.786
*Communication and teamwork	38	56.7	11	64.7	16	55.2	11	52.4	
Leadership	12	17.9	2	11.8	5	17.2	5	23.8	
Decision making	12	17.9	2	11.8	7	24.1	3	14.3	
Situation awareness	5	7.5	2	11.8	1	3.4	2	9.5	
Non- technical skills most needed for team improvement									0.055
*Communication and teamwork	45	67.2	14	82.4	22	75.9	9	42.9	
Decision making	9	13.4	2	11.8	1	3.4	6	28.6	
Situational awareness	8	11.9	1	5.9	4	13.8	3	14.3	
Leadership	5	7.5	0	0	2	6.9	3	14.3	

*Identified as priority training areas

There was an overwhelming positive interest in receiving NTS training in the future with 64 respondents (95.5%) expressing a desire to attend training. Their primary motivation was that NTS training would improve patient safety (66; 98.5%). Table 3 shows the preferred duration and format for NTS training.

**Table 3:** Suggested duration and format of non-technical skills training in the Nigerian context

Ideal duration for NTS training course (*N* = 67)	*n* (%)
<1 day	4 (6.0)
2–3 days	26 (38.8)
1 week	37 (55.2)
**Ideal training format**	
In-person	5 (7.5)
Online (virtual)	15 (22.4)
Blended (virtual and in-person)	47 (70.1)

The most common thematic barriers to respondents attending NTS training in the future included time conflicts (31, 46.3%), and cost barriers (14, 20.9%). Lack of access to training (10, 14.9%) and an unsupportive work environment (9, 13.4%) such as hierarchical dynamics and work-related psychological stress were also identified as key barriers (Table 4). Seventeen respondents (25.4%) identified no barriers to attending future NTS training. The most common enabling factor for attending future NTS training was the desire to improve patient safety (34, 50.7%). The desire for self-improvement (18, 26.9%) and the desire for an improved work environment (10, 14.9%) were also identified as key enabling factors. Five respondents (7.5%) could not highlight any factors which would motivate them to attend a future NTS training course.

**Table 4:** Barriers and enabling factors identified by multidisciplinary teams potentially limiting or enhancing their participation in non-technical skill trainings in Nigeria

	Themes	*n*	(%)	Example phrases
**Barriers**	Hindrances from the perioperative and institutional work environment	40	59.7	Challenges with the “Release by my employer “; “Work stress”; Non-technical skills training may “Clash with normal duties” or be limited by “tight schedule of residency training programme”; “Scheduled time of training coinciding with work”
	Lack of funding to pay for non-technical skills courses	14	20.9	A barrier is “Cost”; “Financial constraints”
	Lack of access to non-technical skills courses, particularly if in-person	10	14.6	Limited access to courses due to the “Insecurity Nigeria is now experiencing”; “Poor Internet services in Nigeria”; “Distance if the program is not online”
**Enabling factors**	The motivation of improved patient safety	34	50.7	“To improve patient outcomes”; “Quest for improvement in surgical outcome”; “Patient safety and satisfaction”; “achieving global excellence for the maximum patient benefit”; “Desire to improve surgical outcome for my patience”; “prevent errors during surgery”
	A drive for self-improvement	18	26.9	Facilitated by a desire “To add value to myself”; “Skill acquisition “; “personal aim at achieving global excellence “; Desire to “To improve my skills and knowledge”
	The motivation of experiencing an improved work environment	10	14.9	Motivated by “reduce toxicity in the Operating Room”; “Strengthening teamwork”; “To see the change in attitude of my colleagues and other workers”; “A better working relationship with other health professionals”; “Improving interpersonal relationships”
	Funding and financial incentives for training	4	6.0	Non-technical skills training would be facilitated by “Sponsorship”; “Scholarship”; “Financial motivation”
	Support from work hierarchy, and removal of workplace barriers	3	4.5	Facilitated by “Convenience”; “Permission from my work place”
	The award of certificates of training or attendance	1	1.5	The award of a formal “certificate” of attendance or other certification in non-technical skills

## Discussion

Most respondents from operating room teams in North-Central Nigeria had never heard of any NTS for surgery framework useful for variable resource contexts and only 8% had ever received any form of NTS training. Interprofessional teams identified communication and teamwork as the most deficient personal skills (38, 57%), and as the most needed for surgical team improvement (45, 67%). There was a very high demand for NTS training by all surgical team members motivated by expectations of improved patient safety and improved interprofessional team dynamics. Week-long, hybrid training courses (with combined in-person and online components) were the preferred format for delivery of NTS education. Factors that would facilitate attendance included a desire for patient safety and self-improvement, while barriers to attendance were conflicts of time, and training costs. The way forward for NTS training in North-Central Nigerian context is interprofessional training in hybrid format which prioritizes communication and teamwork, emphasizes patient safety, and is delivered at low costs.

There is very limited exposure to surgical-team centred NTS frameworks and training in Nigeria, and across West Africa. This is in contrast with the United Kingdom, North America, East Africa, Australasia, Europe, Japan, Malaysia and Sri Lanka, where NOTSS is taught regularly as an integral part of the surgical training programs and continuing medical education [20]. In the Nigerian context, there appears to be emphasis on technical skills over NTS. Over 2,500 Nigerian surgical specialists have been trained by the Nigeria Postgraduate Medical College of Nigeria (NPMCN) and the West African College of Surgeons (WACS), neither of which include structured NTS as a part of their curricula [21–23]. Nursing care training in the context also lacks emphasis on measurable, contextualized NTS [24]. Lack of exposure to these crucial interprofessional skills in regional medical education creates a significant gap in training and practice that needs to be addressed. While systems issues are a major challenge in this context, and much effort goes into handling surgical systems challenges like supply chain, human resources, surgical access, and surgical financing [25], this pragmatic emphasis can lead to a neglect of human factors and NTS. Our survey findings show that poor NTS have been identified as a challenge by interprofessional surgical teams, but training solutions have not yet been identified in the context.

Communication and teamwork were identified as the most important NTSs needed in the Nigerian surgical environment for personal development, team building, and improvement of patient care. The Nigerian health care scene has been a minefield of unhealthy interprofessional rivalry between cadres of health workers [26–28]. This has been responsible for a lack of cooperation, a sense of unwholesome hierarchy, mistrust, and fear that often carries itself into the operating room [27]. These age-old challenges have led to recurrent industrial actions, and counter-industrial actions, organizational tensions [26, 27, 29]. Respondents suggest prioritizing training in communication and teamwork over training in other cognitive aspects of NTS (situation awareness and decision making) as the way forward in the Nigerian context. The Nigerian healthcare system can potentially be enhanced with interprofessional education (IPE) and collaboration [30]. Early, multidisciplinary NTS training is a potential approach to addressing these aspects of the Nigerian surgical, and larger, medical practice space.

Longer-term training was suggested as the ideal format for NTS training in Nigeria. Designing NTS training courses to last for one week, as opposed to a few hours or 2–3 days was strongly suggested by respondents. This might reflect the recognition of the magnitude of exposure necessary to fill the gaps in NTS that have been identified in the context [26, 28]. Although financial incentives rank low as a facilitator, we cannot tell how much this might contribute to the desire for a longer training course. In this context where provider to patient ratio is significantly low [21], it will be challenging to ask clinical providers to leave their clinical duties for a one-week stretch for any type of training. Interval training of two to three days duration twice to thrice a year would be an acceptable compromise to meet provider expectations, while being sensitive to workload, and avoiding the fatigue of an extended course [31].

In-person training appears to have fallen out of favour with respondents as the majority (70.1%) preferred a hybrid approach. This is likely connected with lessons learned by the global community during the COVID-19 pandemic [32]. It is now accepted that high quality education and training can be carried out remotely, via online platforms. Strictly online courses introduce the challenge of wide internet bandwidth, high cost of internet data in Low- and Middle- Income Countries, and the challenges of online learning. The in-person component of blended courses will give the opportunity to include practical, non-didactic components like direct observation and evaluation of learners’ intraoperative NTS in a live operating space for a limited resource setting. Despite the availability of technology solutions including augmented reality, extended virtual reality, and machine learning, they are difficult to implement in a limited resource setting with poor quality internet [33]. Respondents see blended NTS courses as the way forward.

Highest priority barriers included the perioperative and institutional work environment (59.7%), and lack of funding to pay for NTS courses (20.9%). Other courses in Low- and Middle- Income Countries have identified similar barriers [34]. Surgical staff believed that NTS training would “clash with normal duties” or be limited by the “tight schedule of a residency training programme” or that they would not be released to attend trainings by their employers. This can be understood in the light of the low Surgeon Anaesthesia and Obstetrician specialist density in Nigeria (1.8 per 100,000), and the significant impact time away from work for development has on increasing the patient backlog [35]. Attending such courses can easily be seen as disruptions of patient care. Optimizing the work environment, and leadership buy-in are therefore key to preparing surgical staff for a NTS training in this context [36]. Setting up sponsored courses would also encourage engagement. Using mixed methods, Reis et al. found that lack of time, perception of overload at work, inadequate digital infrastructure or competence, and a variety of motivational and emotional elements were barriers to continuing medical education courses among primary care providers [37]. Our findings show that highest priority facilitators were essentially altruistic ideologies undergirding the motivation for NTS training. These include desire for improved patient safety, self-improvement, and improvements in work environment, as opposed to funding and financial incentives or the need for a certificate. Surgeons, anaesthesia providers, and perioperative nurses in the Nigerian context understand the priority of patient safety. Introduction of a multidisciplinary, interprofessional NTS curriculum that is sensitive to these felt needs is important for successful NOTSS-VRC training in this context. Preliminary results have been presented as an abstract at the American College of Surgeons Conference, 2022 [38].

## Limitations

Although this work sampled surgical service providers at secondary and tertiary level facilities, some surgical services (circumcision, debridement, initial open fracture care, incision, and drainage of abscesses etc.), are also being provided at primary level in Nigeria and other parts of sub-Saharan Africa and were not included in this survey. In addition, this survey was purposively limited to North-Central Nigeria due to maximize resources and connections. It could have been distributed to a broader population for more representative national data. Further studies involving providers at this level may provide a more holistic understanding of motivations for NTS training. Secondly, challenges of internet access in an LMIC like Nigeria might bias results, as only surgical providers, anaesthesiologists, and nurses with internet access or internet enabled devices could have responded. The likelihood of exposure to NTS may be higher among those with internet enabled devices, considering the increase in online education following the COVID-19 pandemic. Future research should consider hybrid online and interviewer-administered paper surveys to ensure a more representative sample.

## Conclusions

Surgical teams in North-Central Nigeria are highly motivated for NTS training. Multi-disciplinary and inter professional teams consisting of perioperative nurses, anaesthesia providers, and surgeons believe that NTS skill development can improve patient safety, team dynamics, and personal performance. In the context of Nigeria, implementation should emphasize communication and teamwork to address the tensions and interprofessional rivalry noted in the local work culture. A hybrid, low-cost approach to training (combining online and in-person components) is preferred by respondents. Optimizing the work environment and ensuring that hospital and theatre leadership teams buy into the programs and champion NTS training are key to successful NOTSS-VRC training in this context. The way forward for NTS training in North-Central Nigeria is hybrid, low cost, inter professional training with an emphasis on teamwork and communication for improved patient outcomes and surgical safety.

## Data Availability

The dataset used and/or analysed during the current study are available from the corresponding author on reasonable request.

## Abbreviations

IPE: Interprofessional education
NTS: Non-technical skills
NOTSS: Non-Technical Skills for Surgeons
NOTSS-VRC: Non-Technical Skills for Surgery in Variable Resource Context
CHERRIES: Checklist for Reporting Results of Internet E-Surveys
